# Effect Modifiers of Graded Sensorimotor Retraining for Chronic Low Back Pain: A Secondary Analysis of the RESOLVE Randomized Trial

**DOI:** 10.1001/jamanetworkopen.2025.52787

**Published:** 2026-01-13

**Authors:** Martjie Venter, James H. McAuley, Harrison J. Hansford, Mia Ryan, Matthew K. Bagg, Benedict M. Wand, Aidan G. Cashin

**Affiliations:** 1 School of Health Sciences, Faculty of Medicine and Health, University of New South Wales Sydney, Kensington, New South Wales, Australia; 2 Centre for Pain IMPACT, Neuroscience Research Australia, Randwick, New South Wales, Australia; 3 School of Health Sciences, The University of Notre Dame Australia, Fremantle, Western Australia, Australia

## Abstract

**Importance:**

Outcomes for people with low back pain (LBP) may be improved through targeting treatments to subgroups with particular experiences, characteristics, or symptoms and clinical factors.

**Objective:**

To investigate potential treatment effect modifiers of graded sensorimotor retraining affecting pain intensity and disability level in the short and long terms for people with chronic LBP.

**Design, Setting, and Participants:**

This post hoc exploratory secondary analysis of the RESOLVE randomized clinical trial in Sydney, Australia, was conducted from November 5, 2024, to May 27, 2025. Trial participants were recruited from primary care settings and randomly allocated (1:1) to receive either graded sensorimotor retraining (treatment group) or attention control and sham procedures (sham control group). Eligible participants were adults aged 18 to 70 years who reported chronic nonspecific LBP (lasting ≥12 weeks), with or without leg pain, that was rated at least a 3 out of 10 in pain intensity. Statistical analyses were conducted from November 11 to December 6, 2024.

**Interventions:**

The treatment group completed 12 weekly clinical sessions of the graded sensorimotor retraining package, which consisted of pain science education, premovement training, and graded movement and loading. The sham control group completed 12 weekly sessions, without advice or education, of sham electrotherapy to the back and sham noninvasive brain stimulation.

**Main Outcomes and Outcome Measures:**

Primary outcomes were pain intensity (measured with the 11-point Numerical Rating Scale ranging from 0 [no pain] to 10 [worst imaginable pain]) and disability level (measured with the 24-item Roland-Morris Disability Questionnaire with scores ranging from 0-24 [higher scores indicating greater levels of disability]) assessed at 18 weeks and 52 weeks after randomization. A formal moderation analysis was performed using a test for statistical interaction. Eight baseline variables—psychoactive medication use, pain intensity, disability level, beliefs about back pain consequences, kinesiophobia, pain catastrophizing, pain self-efficacy, and back perception—were investigated for their potential treatment effect modification.

**Results:**

The study included 276 participants (mean [SD] age, 46 [14.3] years; 138 females [50.0%]), 138 of whom were randomized to the treatment group and 138 of whom were randomized to the sham control group. Pain self-efficacy, pain catastrophizing, pain intensity, and psychoactive medication use showed no evidence of modifying the effect of the intervention. Impaired back perception was identified as a potential treatment effect modifier of pain intensity (β-coefficient = 0.18 [95% CI, 0.05-0.32]; *P* = .007) at the 52-week follow-up time point. Hypothesis-generating evidence (*P* < .20) indicated potential effect modification by kinesiophobia (on pain intensity at 18-week follow-up: β-coefficient = 0.06 [95% CI, −0.02 to 0.14], *P* = .15; 52-week follow-up: β-coefficient = 0.07 [95% CI, −0.02 to 0.16], *P* = .12), baseline disability level (on disability level at 18-week follow-up: β-coefficient = −0.15 [95% CI, −0.38 to 0.07], *P* = .17), beliefs about back pain consequences (on disability level at 52-week follow-up: β-coefficient = 0.16 [95% CI, −0.05 to 0.37], *P* = .14) and back perception (on pain intensity at 18-week follow-up: β-coefficient = 0.10 [95% CI, −0.02 to 0.22], *P* = .09; on disability level at 18-week follow-up: β-coefficient = 0.22 [95% CI, −0.04 to 0.48], *P* = .10; 52-week follow-up: β-coefficient = 0.27 [95% CI, 0.00-0.55], *P* = .05).

**Conclusions and Relevance:**

This secondary analysis found that the benefits of graded sensorimotor retraining are likely to be similar for all people with chronic nonspecific LBP presenting for care. Future clinical trials are needed to further explore and assess the role of the potential treatment effect modifiers identified in this analysis.

**Trial Registration:**

ANZCTR Identifier: ACTRN12615000610538

## Key Points

**Question:**

Which variables modify the effect of graded sensorimotor retraining on pain intensity and disability level at 18 weeks and 52 weeks of follow-up?

**Findings:**

In this secondary analysis of a randomized clinical trial involving 276 participants, there was limited evidence of treatment effect modification by the 8 baseline variables identified. Back perception was identified as a potential treatment effect modifier for pain intensity at the 52-week follow-up time point.

**Meaning:**

These findings suggest that the benefits of graded sensorimotor retraining may be similar for all people with chronic low back pain, regardless of individual differences at baseline.

## Teaser

This secondary analysis of a randomized clinical trial assesses changes in pain intensity and disability level associated with 8 treatment effect modifiers among people with chronic low back pain.

## Introduction

Low back pain (LBP) contributes the largest share of the disability burden worldwide.^1^ In 2020, LBP affected 619 million people globally,^1^ with one-third of this population likely to report chronic, ongoing pain and disability for more than 3 months after symptom onset.^2^ People with chronic LBP have a poor prognosis^3,4^ and account for a disproportionately large percentage of the burden associated with LBP due to ongoing treatment costs, loss of work productivity, and reduced quality of life.^5,6^

A top priority in LBP research is to identify effective and cost-effective treatments for subgroups of individuals who may have differential treatment responses.^7,8,9^ These subgroups comprise patients who share characteristics such as symptoms or prognostic factors.^10^ The targeted use of effective treatments for certain subgroups of people with chronic LBP has the potential to improve treatment outcomes. A new theoretically informed treatment for chronic LBP—graded sensorimotor retraining^11^—demonstrated clinically meaningful and sustained improvements in pain intensity and disability level when investigated in a sham- and attention-controlled randomized clinical trial.^12^ Although graded sensorimotor retraining was effective, it remains unclear whether certain baseline characteristics may modify the treatment effect (effect modification).

There is a need to investigate variables that modify the effect (also known as moderators) of graded sensorimotor retraining both to optimize the treatment and to facilitate its implementation in practice for people with chronic LBP.^13^ By identifying baseline characteristics associated with treatment response, the results may guide the refinement of the intervention to target less responsive groups and facilitate delivery in clinical settings to people more likely to benefit. Guidelines for treatment effect modification studies state that the hypothesized moderators should be prespecified based on sound theoretical support and relevant expert consultation.^14,15^ This study aimed to investigate potential treatment effect modifiers of graded sensorimotor retraining affecting pain intensity and disability level in the short and long terms for people with chronic LBP.

## Methods

### Study Design, Setting, and Participants

This post hoc secondary analysis was declared in a statistical analysis plan^16^ (Supplement 1) after completion of the RESOLVE trial, a 2-group parallel, sham-controlled randomized clinical trial conducted at a medical research institute in Sydney, Australia.^12^ We performed a moderation analysis using data from this trial. The trial protocol^17^ and statistical analysis plan^18^ outlining the methods were published previously, as were the main findings,^12^ mediation analysis,^19^ and qualitative analyses,^20,21^ providing detailed information on the trial design, procedures, and findings. The University of New South Wales Human Research Ethics Committee approved the trial. All participants provided written informed consent prior to trial enrollment.

Participants were recruited by direct referral from primary care clinicians and from the community through local and online advertisements. Eligible participants were adults between 18 and 70 years of age who reported chronic nonspecific LBP (lasting ≥12 consecutive weeks), with or without associated leg pain, that was rated a minimum of 3 out of 10 on an 11-point Numerical Rating Scale (NRS). Additionally, participants were required to speak fluent English, have internet access, and have a trusted person to assist them with the home components of the intervention. Participants were excluded if they had radicular pain; had LBP caused by a serious medical condition (infection, fracture, or malignant neoplasm); were pregnant or gave birth in the previous 6 months; had undergone spinal surgery in the past 12 months or were scheduled for major surgery in the upcoming 12 months; had an uncontrolled mental health condition that would prevent participation; and had any other contraindications to physical activity, transcranial direct current stimulation, cranial electrical stimulation, low-intensity laser therapy, or short-wave diathermy.

Participants were randomized between December 10, 2015, and July 25, 2019, and follow-up was completed by February 3, 2020. This secondary analysis was conducted from November 5, 2024, to May 27, 2025.

### Randomization and Blinding

Participants were randomly allocated (1:1) through sealed, opaque, sequentially numbered envelopes to receive either graded sensorimotor retraining (treatment group) or the matched attention control and sham procedures (sham control group). Random allocation occurred immediately after baseline assessment when the clinician opened the next numbered envelope. The randomization sequence was generated using a computer-generated block randomization (random block size range, 2-10). Participants as well as the researchers collecting and analyzing outcome data were blinded to the group allocation. To maintain blinding, participants were informed that both interventions targeted the central nervous system and back function, and no trial information was publicly disclosed. The trial clinicians remained unblinded.

### Interventions

Both treatment and control interventions consisted of 12 in-person sessions, each lasting 1 hour, that were delivered by trained study clinicians. Participants were required to complete a home treatment program approximately 5 times per week for 30 minutes. Detailed intervention descriptions can be found in the published trial findings^12^ and protocol.^17^

#### Graded Sensorimotor Retraining 

Graded sensorimotor retraining is a rehabilitation package developed from the theoretical framework described in the fit-for-purpose model,^11^ which evolved from the maladaptive perceptions model.^22,23^ This intervention package integrates approaches (pain science education, premovement training, and graded movement and loading) that have a clinically meaningful effect on pain and disability.^24,25,26^ The first component of the package aims to help the person understand that it is safe and helpful to move. This educational component is delivered across the 12 sessions and focuses on contemporary pain science.^24^ The second component aims to refine neural representations of the back and the precision of sensorimotor processing so that the back feels safe to move. This aim is achieved through premovement training,^25^ which incorporates both tactile precision practice and mental rehearsal of movement through graded motor imagery as well as active exercises designed to improve proprioceptive precision. The third component aims to load the back to promote beneficial tissue adaptations and enable the individual to experience safety with movement of increasing complexity and load. This aim is achieved through precision-focused and feedback-enriched graded movement and loading until return to functional goals.^26,27^

#### Attention Control and Sham Procedures 

Attention control and sham procedures involved discussion of the participant’s back pain experience without advice or education provided, sham laser therapy to the most painful area of the back, sham shortwave diathermy to the back, and sham transcranial direct current stimulation applied to the motor and prefrontal cortices on the contralateral side of the worst pain. Participants were provided with a sham cranial electrical device to use at home. The intervention was designed to control for time with an expert clinician, graded progression of treatment in the active intervention and expectation of a central nervous system–directed component of care (as outlined in the trial protocol and participant information sheet), and matching the active intervention’s home component.

### Outcome Measures

The primary outcomes for this study were pain intensity (measured with the 11-point NRS, with a score range of 0 [indicating no pain] to 10 [indicating worst imaginable pain]) and disability level (measured with the 24-item Roland-Morris Disability Questionnaire [RMDQ], with a scale range of 0-24 [higher scores indicating greater levels of disability]) assessed at 18 weeks and 52 weeks after randomization. These outcomes were included because they represent distinct dimensions of LBP, align with established core outcome sets for LBP,^28^ and reflect the priorities identified by people with a lived experience.^29^

### Potential Treatment Effect Modifiers

Prior to randomization, baseline characteristics were collected from participants using self-reported questionnaires. Eight potential treatment effect modifiers were selected—psychoactive medication use,^30,31,32,33,34^ pain intensity,^31,35,36,37^ disability level,^31,37,38,39^ beliefs about back pain consequences,^19,40^ kinesiophobia,^41^ pain catastrophizing,^19^ pain self-efficacy,^19,40,42^ and back perception^22^—that represent common clinical and psychosocial characteristics observed in populations with chronic LBP. These variables were selected based on prior evidence of moderation or their theoretical relevance to the graded sensorimotor retraining intervention. Table 1 describes the 8 potential treatment effect modifiers as well as the assessment method, supporting evidence, rationale, and hypothesized direction for each moderator variable. We selected these effect modifiers and specified their hypothesized direction in the statistical analysis plan prior to executing the moderation analysis.^10,15,16,43^

**Table 1.** Potential Treatment Effect Modifiers

Treatment effect modifier	Variable assessment	Supporting evidence	Rationale	Hypothesized direction
Psychoactive medication use	Dichotomous (yes, no)— “Have you taken any medication prescribed by a GP or specialist doctor for your back pain?” *(Please list all medications taken in the past month)*— where yes entails any incident or ongoing script for any psychoactive medicine	Beneciuk et al,^30^ 2017; Gurung et al,^31^ 2015; Beneciuk et al,^32^ 2023; Hayden et al,^33^ 2020; Venter et al,^34^ 2024	Psychoactive medication use affects CNS processing, which may influence a person’s capacity to engage with the intervention	No medication use > medication use
Pain intensity	Continuous (scale: 0-10)— “In the past week, on average, how intense was your pain on a 0-10 scale where 0 is ‘no pain’ and 10 is ‘pain as bad as it could be’?”	Gurung et al,^31^ 2015; de Zoete et al,^35^ 2021; Hahne et al,^36^ 2017; Holden et al,^37^ 2023	People with more severe pain likely have more pronounced sensorimotor disruption and maladaptive pain processing, providing greater potential for an improved response to treatment	Higher baseline pain intensity > lower baseline pain intensity
Disability level	Continuous— Roland-Morris Disability Questionnaire (scale: 0-24, with higher scores indicating higher levels of disability)	Gurung et al,^31^ 2015; Hee et al,^38^ 2021; Holden et al,^37^ 2023; Hancock et al,^39^ 2024	People with more functional limitations may respond better to a treatment aimed at restoring movement confidence and control	Higher baseline disability level (ie, poorer physical function) > lower baseline disability level
Beliefs about back pain consequences	Continuous— Back Beliefs Questionnaire (scale: 9-45, with lower scores indicating more negative beliefs about the consequences of back pain)	Cashin et al,^19^ 2023; Chen et al,^40^ 2023	The intervention challenges maladaptive beliefs about the back pain consequences; therefore, those with negative beliefs about back pain consequences may respond better	Negative beliefs about back pain consequences > positive beliefs about back pain consequences
Kinesiophobia	Continuous— Tampa Scale of Kinesiophobia (scale: 17-68, with higher scores indicating an increasing degree of kinesiophobia)	Murillo et al,^41^ 2023	The intervention progressively exposes individuals to feared movements, targeting movement-related fear; therefore, those with worse fear of movement may respond better	Higher kinesiophobia > lower kinesiophobia
Pain catastrophizing	Continuous— Pain Catastrophizing Scale (scale: 0-25, with higher scores indicating more severe pain catastrophizing)	Cashin et al,^19^ 2023	Higher catastrophizing reflects stronger maladaptive pain cognitions, which the intervention targets; therefore, those with higher pain catastrophizing may respond better	Higher pain catastrophizing > lower levels of pain catastrophizing
Pain self-efficacy	Continuous— Pain Self-Efficacy Questionnaire (scale: 0-60, with higher scores indicating more confidence in managing one’s pain)	Roseen et al,^42^ 2021; Cashin et al,^19^ 2023; Chen et al,^40^ 2023	Greater pain self-efficacy may facilitate better engagement with and adherence to the intervention; therefore, these people may respond better	Higher pain self-efficacy > lower pain self-efficacy
Back perception	Continuous— Fremantle Back Awareness Questionnaire (scale: 0-36, with higher scores indicating higher levels of disrupted body image)	Wand et al,^22^ 2016	The intervention targets disrupted back perceptions; therefore, those with higher levels of body perception disturbances may respond better	Higher levels of body perception disturbance > lower levels of body perception disturbance

Abbreviations: CNS, central nervous system; GP, general practitioner.

### Statistical Analysis

#### Main Analysis

Baseline demographic and clinical characteristics were summarized using counts (percentages) for categorical variables and means (SDs) or medians (IQRs) for continuous variables. We conducted a formal moderation analysis using a test for statistical interaction.^14,43^ We used separate linear regression models at the 18-week and 52-week follow-up time points to evaluate a potential moderator variable against each outcome by incorporating a group-moderator interaction term in each model. We examined residual plots to assess linearity, normality, and homoscedasticity; leverage and influence plots to detect influential outliers; and scatterplots to check for collinearity. We identified no threat to the assumptions for each model. Each model was constructed using a term for outcome (pain intensity or disability level), group allocation, and potential moderator and an interaction term for group and moderator. We estimated the effect size and 95% CI and reported them alongside the *P* value for each group-moderator interaction. The moderation effect of the dichotomous moderator variable (ie, psychoactive medication use) was interpreted as the difference in intervention effect (treatment vs sham control) between participants who used and did not use medication. The moderation effect of the continuous moderator variables was interpreted as the difference in intervention effect (treatment vs sham control) for a single unit change in the moderator. We considered *P* < .20 as hypothesis generating and *P* < .05 as statistically significant, similar to previous studies investigating treatment effect modifiers.^30,31^ Statistical analyses were conducted from November 11 to December 6, 2024, using R, version 4.4.2 (R Project for Statistical Computing).^44^

#### Missing Data

We assessed the proportions and patterns of missing data for the moderator and outcome variables and performed multiple imputation by chained equations^45^ for 10 datasets using the mice package^46^ in R, version 4.4.2.^44^ The imputation model included baseline covariates (age, sex, back pain duration, other areas of pain, work absence, compensable status, educational level, depressive symptoms score, and Insomnia Severity Index), group allocation, outcome variables at the 18-week and 52-week follow-up time points, and all potential moderator variables. Estimates were pooled using Rubin rules,^47^ and all reported results were based on the pooled imputed estimates.

## Results

### Sample Characteristics

A total of 276 participants were enrolled in the RESOLVE trial, 138 of whom were randomized to the treatment group and 138 of whom were randomized to the sham control group. Figure shows the flow of trial participants.

**Figure.**
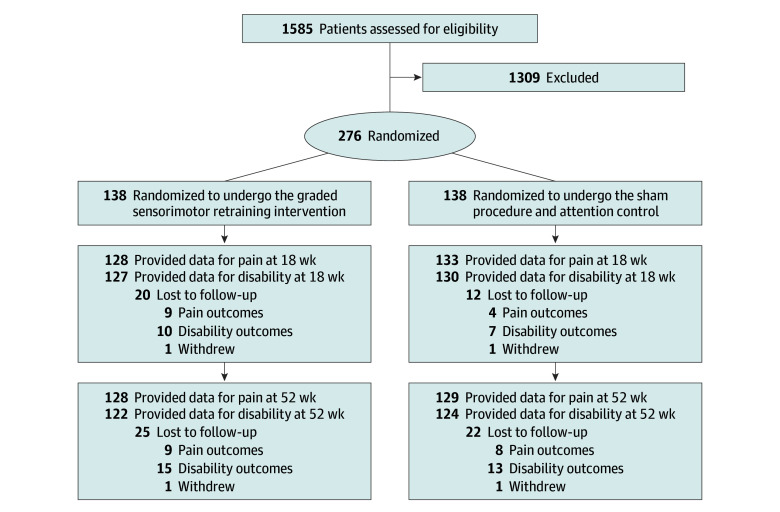
RESOLVE Trial Participant Flowchart

Baseline characteristics for included participants are summarized in Table 2. The mean (SD) age was 46 (14.3) years; there were 138 females (50.0%) and 138 males (50.0%); and 154 participants (55.8%) had a university-level degree. At baseline, participants had moderate to high pain intensity scores (mean [SD] NRS score, 5.7 [1.8]) and moderate disability levels (mean [SD] RMDQ score, 9.8 [5.2]). There were no missing data for the moderator variables. Proportions of missing data for the outcome variables were 5.4% at 18 weeks and 6.9% at 52 weeks of follow-up for pain intensity and 6.9% at 18 weeks and 10.9% at 52 weeks of follow-up for disability level.

**Table 2.** Baseline Characteristics of Participants (N = 276)^a^

Characteristic	Participant group	
	Treatment (n = 138)	Sham control (n = 138)
Age, y	44.7 (14.5)	47.0 (14.1)
Sex, No. (%)		
Female	72 (52.2)	66 (47.8)
Male	66 (47.8)	72 (52.2)
Educational level, No. (%)^b^		
University-level degree	77 (55.8)	77 (56.2)
Diploma	19 (13.8)	20 (14.6)
Vocational certificate	17 (12.3)	18 (13.1)
Year 12	18 (13.0)	10 (7.3)
Year 10	7 (5.1)	12 (8.8)
Duration of current episode of LBP, median (IQR), y^c^	5 (4-10)	6 (3-11)
Work absence or reduction, No. (%)	27 (19.6)	26 (18.8)
Compensation claimed, No. (%)	9 (6.5)	17 (12.3)
Insomnia Severity Index^d^	13.5 (7.7)	14.0 (7.5)
Depressive symptoms score^e^	4.6 (5.0)	5.0 (4.9)
Potential treatment effect modifiers		
Pain intensity^f^	5.6 (1.8)	5.8 (1.8)
Disability level^g^	9.6 (5.4)	10 (5.0)
Psychoactive medication use, No. (%)	28 (20.3)	36 (26.1)
Pain catastrophizing^h^	19.1 (12.7)	21 (12.8)
Beliefs about back pain consequences^i^	26.2 (6.6)	25.6 (6.4)
Kinesiophobia^j^	38.5 (7.6)	38.4 (7.3)
Pain self-efficacy^k^	40.4 (13.7)	38.7 (13.0)
Back perception score^l^	6.96 (5.0)	7.91 (4.7)

Abbreviation: LBP, low back pain.

^a^ Data are presented as the mean (SD) unless indicated otherwise.

^b^ Data were missing for 1 participant in the sham control group.

^c^ Data were missing for 1 participant in the treatment group and 6 participants in the sham control group.

^d^ Insomnia Severity Index scale range: 0 to 28, with higher scores indicating worse sleep quality.

^e^ Subscale from the Depression, Anxiety, and Stress Scale range: 0 to 21, with higher scores indicating more depressive symptoms.

^f^ Eleven-point Numerical Rating Scale range 0 to 10, with higher scores indicating higher pain intensity.

^g^ Roland-Morris Disability Questionnaire scale range: 0 to 24, with higher scores indicating higher levels of disability.

^h^ Pain Catastrophizing Scale range: 0 to 25, with higher scores indicating more severe pain catastrophizing.

^i^ Back Beliefs Questionnaire scale range: 9 to 45, with lower scores indicating more negative beliefs about the consequences of back pain.

^j^ Tampa Scale of Kinesiophobia scale range: 17 to 68, with higher scores indicating an increasing degree of kinesiophobia.

^k^ Pain Self-Efficacy Questionnaire scale range: 0 to 60, with higher scores indicating more confidence in managing one’s pain.

^l^ Fremantle Back Awareness Questionnaire scale range: 0 to 36, with higher scores indicating higher levels of disrupted body image.

### Treatment Effect Modifiers

Table 3 reports the effect size and statistical significance of the group-moderator interactions between each of the 8 investigated moderator variables and pain intensity and disability level at 18 weeks and 52 weeks of follow-up. There was no evidence that baseline psychoactive medication use, pain intensity, disability level, pain catastrophizing, and pain self-efficacy modified the effect of the intervention (graded sensorimotor retraining vs attention control and sham procedures) on pain intensity at the 18-week or 52-week follow-up time points. Similarly, there was no evidence that baseline psychoactive medication use, pain intensity, beliefs about back pain consequences, kinesiophobia, pain catastrophizing, and pain self-efficacy modified the effect of the intervention on disability level at 18 weeks after randomization. Baseline psychoactive medication use, pain intensity, disability level, kinesiophobia, pain catastrophizing, and pain self-efficacy also did not modify the intervention effect on disability level at 52 weeks after randomization.

**Table 3.** Treatment Effect Modification on Pain Intensity and Disability Level

Potential treatment effect modifier	Pain intensity				Disability level			
	18-wk Follow-up		52-wk Follow-up		18-wk Follow-up		52-wk Follow-up	
	Interaction coefficient (95% CI)^a^	*P* value	Interaction coefficient (95% CI)^a^	*P* value	Interaction coefficient (95% CI)^a^	*P* value	Interaction coefficient (95% CI)^a^	*P* value
Psychoactive medication use (yes/no)	0.19 (−1.21 to 1.60)	.79	−0.04 (−1.58 to 1.50)	.96	1.43 (−1.58 to 4.45)	.35	0.16 (−2.91 to 3.22)	.92
Pain intensity (scale: 0-10)	−0.16 (−0.47 to 0.15)	.31	−0.13 (−0.46 to 0.21)	.46	−0.29 (−0.99 to 0.40)	.41	−0.37 (−1.11 to 0.36)	.32
Disability level (scale: 0-24)	0.02 (−0.01 to 0.13)	.72	0.04 (−0.08 to 0.17)	.48	−0.15 (−0.38 to 0.07)	.17	0.13 (−0.37 to 0.10)	.26
Beliefs about back pain consequences (scale: 9-45)	−0.04 (−0.14 to 0.05)	.37	−0.04 (−0.14 to 0.05)	.39	0.06 (−0.14 to 0.26)	.57	0.16 (−0.05 to 0.37)	.14
Kinesiophobia (scale: 17-68)	0.06 (−0.02 to 0.14)	.15	0.07 (−0.02 to 0.16)	.12	−0.01 (−0.18 to 0.16)	.92	−0.02 (−0.20 to 0.15)	.79
Pain catastrophizing (scale: 0-52)	0.02 (−0.03 to 0.06)	.51	0.01 (−0.04 to 0.06)	.69	0.01 (−0.01 to 0.12)	.85	0.00 (−0.10 to 0.10)	.93
Pain self-efficacy (scale: 0-60)	0.00 (−0.05 to 0.04)	.86	0.00 (−0.04 to 0.05)	.85	0.03 (−0.06 to 0.12)	.47	0.04 (−0.06 to 0.13)	.46
Back perception (scale: 0-36)	0.10 (−0.02 to 0.22)	.09	0.18 (0.05 to 0.32)	.007	0.22 (−0.04 to 0.48)	.10	0.27 (0.00 to 0.55)	.05

^a^ The coefficients are the interaction terms and 95% CIs from linear regression models representing the difference in treatment effect of graded sensorimotor retraining vs attention control and sham procedures for a single unit change in the baseline moderator score (continuous variables) or between participants who used and did not use psychoactive medication (dichotomous variable). Negative coefficients represent greater treatment effect of graded sensorimotor retraining vs attention control and sham procedures for each unit of higher score on the moderator at baseline (continuous variables) or between participants who used and did not use psychoactive medication (dichotomous variable).

The effect of the intervention on pain intensity was modified by back perception at 52 weeks of follow-up (β-coefficient = 0.18 [95% CI, 0.05-0.32]; *P* = .007) (Table 3). People with higher levels of disrupted back perception at baseline experienced less benefit from graded sensorimotor retraining at the 52-week follow-up time point.

### Hypothesis-Generating Evidence for Treatment Modification

Hypothesis-generating evidence showed that higher levels of kinesiophobia may modify the effect of the intervention on pain intensity at the 18-week (β-coefficient = 0.06 [95% CI, −0.02 to 0.14]; *P* = .15) and 52-week (β-coefficient = 0.07 [95% CI, −0.02 to 0.16]; *P* = .12) follow-up time points; however, the 95% CIs included no moderating effect. Lower levels of disability at baseline may modify the effect of the intervention on disability level at 18 weeks (β-coefficient = −0.15 [95% CI, −0.38 to 0.07]; *P* = .17), and positive beliefs about back pain consequences may modify the effect of the intervention on disability level at 52 weeks of follow-up (β-coefficient = 0.16 [95% CI, −0.05 to 0.37]; *P* = .14); however, the 95% CIs included no moderating effect. Finally, higher levels of disrupted back perception may modify the effect of the intervention on pain intensity and disability level at the 18-week (β-coefficient = 0.10 [95% CI, −0.02 to 0.22; *P* = .09] and 0.22 [95% CI, −0.04 to 0.48; *P* = .10]) and disability level at the 52-week (β-coefficient = 0.27 [95% CI, 0.00-0.55]; *P* = .05) follow-up time points; however, the 95% CIs included no moderating effect.

## Discussion

This exploratory secondary analysis of data from the RESOLVE trial sought subgroups of individuals who might respond differently to graded sensorimotor retraining compared with attention control and sham procedures. We found limited evidence of any prominent treatment effect modifiers. Participants presenting with chronic LBP and lower levels of disrupted back perceptions may benefit more from graded sensorimotor retraining in the long term. Our findings suggest the benefits of graded sensorimotor retraining are likely to be similar for people presenting with typical characteristics of chronic nonspecific LBP.

To our knowledge, back perception has not been investigated as a treatment effect modifier for people with chronic LBP. The development of graded sensorimotor retraining was informed by evidence that back perceptions may become disrupted following nociplastic changes associated with chronic LBP,^48^ which can contribute to and maintain pain,^22,49^ and that improvements in back perception are associated with reduced pain and disability.^25,50^ Counter to our hypothesis, people with less disrupted back perceptions experienced greater benefits with graded sensorimotor retraining. A reason for this finding may be that individuals with higher levels of body perception disturbance found it difficult to engage with the intervention effectively (particularly the premovement tasks) or that the ability of the intervention to affect back perception remains uncertain.^19^ This finding that back perception was a treatment effect modifier requires replication to determine whether the observed result was spurious or might instead reflect the measurement tool used (Fremantle Back Awareness Questionnaire^22^), which has not undergone adequate content validation; trial sample characteristics (eg, low levels of [ie, better] back perception); or a surrogate for another unmeasured factor (eg, emotional regulation), which may be more directly associated with the outcome of interest. An investigation of the role of back perceptions in other brain- and body-directed treatments^51,52^ for LBP may help guide the targeting of these interventions to relevant patient groups.

Lower disability levels, positive beliefs about back pain consequences, and higher levels of kinesiophobia at baseline were identified as possible treatment effect modifiers. We considered *P* < .20 as hypothesis-generating evidence of effect modification, identifying factors that may be worth investigating in future studies with larger sample sizes. Future trials should measure and continue to review the role of moderator variables as subgroups that may affect treatment responses. However, for this hypothesis-generating evidence (*P* < .20), the 95% CI included no moderation effect; therefore, these results need to be interpreted with caution.

This secondary analysis provided evidence to move the implementation of graded sensorimotor retraining from a research setting to clinical practice. The treatment appears to be suitable for people with chronic nonspecific LBP in primary care. Assessing the back perceptions of patients presenting with chronic LBP using validated tools (eg, Fremantle Back Awareness Questionnaire^22^) may be informative for identifying those who may not experience as great a benefit and for guiding treatment decisions. For example, clinicians should be mindful that people with high scores on the Fremantle Back Awareness Questionnaire may require extra support throughout the course of the intervention, beyond what is currently provided in the intervention. Extra support may include augmenting sensory feedback^53^ (eg, virtual reality or biofeedback), slowing task progression, or increasing premovement training before progressing to movement tasks. Back perception could also be considered in future trial design, particularly when making decisions about participant selection criteria,^54^ and for inclusion in future clinical prediction tools.^55^ Finally, future randomized clinical trials should be designed with larger sample sizes to statistically accommodate for and prospectively plan moderation analyses.

### Strengths and Limitations

This study has several strengths. First, we used data from the RESOLVE trial, a high-quality sham-controlled randomized clinical trial with limited missing data.^12^ Second, we followed methodological guidelines for studies investigating treatment effect modifiers, including selecting a limited set of theory-informed variables prior to executing the analysis.^14,15^ Third, we evaluated all continuous treatment effect modifier variables in their continuous form, rather than dichotomizing them. Fourth, the baseline clinical and demographic characteristics of the study sample were comparable with those of patients with chronic LBP, suggesting that the results are generalizable.

This study also has some limitations. First, the RESOLVE trial was not powered for a moderation analysis, which is common in the effect modification literature.^14^ Second, the treatment effect modification on pain intensity and disability level, although statistically significant and clinically meaningful, was small and moderate, respectively,^56^ which may reduce the plausibility for finding significant moderators. Third, the sham control comparison may have limited our ability to detect treatment moderators, given that both groups likely engaged active mechanisms, masking subgroup differences. Fourth, we investigated a single variable at a time, which may affect the ability of findings to directly inform clinical decision-making because patients may have multiple varying characteristics.

## Conclusions

This study found limited evidence of subgroups of people with chronic LBP who respond differently to graded sensorimotor retraining vs attention control and sham procedures. Similar benefits are likely for all people with chronic nonspecific LBP. Future clinical trials are needed to explore the treatment effect modifiers identified in this study and to assess their role in LBP management.

## Notes

Supplement 1.
Statistical Analysis Plan

Supplement 2.
Data Sharing Statement
