# Supplementary material for: Effect Modifiers of Graded Sensorimotor Retraining for Chronic Low Back Pain: A Secondary Analysis of the RESOLVE Randomized Trial
Source: JAMA Netw Open. 2026 Jan 13;9(1):e2552787. doi: 10.1001/jamanetworkopen.2025.52787 (PMC12801085; doi:10.1001/jamanetworkopen.2025.52787)
Supplement: Supplement 1. — Statistical Analysis Plan [file jamanetwopen-e2552787-s001.pdf]

Moderation analysis of the RESOLVE trial: statistical analysis plan

Clinical trials registration number: ACTRN12615000610538
SAP version: 1st version
Date: 31st October 2024

This document is a statistical analysis plan for a moderation analysis of the RESOLVE trial. Detailed information on the RESOLVE trial can be found in the following article:

Bagg MK, Wand BM, Cashin AG, et al. Effect of Graded Sensorimotor Retraining on Pain Intensity in Patients With Chronic Low Back Pain: A Randomized Clinical Trial. *JAMA.* 2022;328(5):430–439. doi:10.1001/jama.2022.9930

Contributors:
Martjie Venter[1,2], James H McAuley[1,2], Matthew K Bagg[1,3,4], Benedict M Wand[3], Aidan G Cashin[1,2]

[1] Centre for Pain IMPACT, Neuroscience Research Australia, Australia
[2] School of Health Sciences, University of New South Wales, Australia
[3] School of Health Sciences, The University of Notre Dame Australia, Fremantle, Australia.
[4] Perron Institute for Neurological and Translational Science, Nedlands, Western Australia

The RESOLVE trial (1) was a two-group parallel randomised controlled trial evaluating the effectiveness of education and graded sensorimotor retraining compared to an attention control with sham procedures on pain intensity and disability among 276 participants with chronic non-specific low back pain. The RESOLVE trial showed that education plus graded sensorimotor retraining compared to the attention control with sham procedures significantly improved pain intensity and disability at 18 weeks (primary timepoint). It is important to assess whether there are any subgroups that may respond differently to this intervention. This statistical analysis plan describes the planned moderation analysis of the RESOLVE trial.

Primary objective

The primary objective in this study is to assess any moderation of the education and graded sensorimotor retraining intervention effects on pain intensity and disability at 18-weeks post randomisation by considering a set of plausible baseline participant characteristics. We aim to identify the characteristics of participants with chronic non-specific low back pain who are more or less likely to benefit from education and graded sensorimotor retraining.

Secondary objective

The secondary objective in this study is to assess moderation of the education and graded sensorimotor retraining intervention effects on pain intensity and disability at the 52-week time point.

Outcomes

The outcomes of interest for this study are pain intensity, assessed using an 11-point numerical rating scale, and low back pain related disability, assessed using the 24-item Roland Morris Disability Questionnaire, both collected at 18- and 52-weeks post randomisation.

Moderators

The selection of potential treatment effect modifiers for this study was based on published literature investigating treatment moderators in musculoskeletal disorders, prognostic cohort studies, and theoretical support. Potential treatment effect modifiers are presented in Table 1.

Table 1. Potential treatment effect modifiers

Potential treatment effect modifier	Variable assessment	Variable operationalisation	Evidence supporting potential effect modification	Hypothesized direction of effect modification
Psychoactive medication use	**Dichotomous**<br>"Have you taken any medication prescribed by a GP or specialist doctor for your **back pain**?"<br><br>(Please list all medications taken in the past month)	**Dichotomous**<br>Yes<br>No<br><br>Where 'Yes' entails any incident or ongoing script for any psychoactive medicine	Venter et al (2024)(6)<br>Beneciuk et al (2017) (7)<br>Gurung et al (2015) (8)<br>Beneciuk et al (2023) (9)<br>Hayden et al (2020) (10)	No medication use > medication use
Baseline pain intensity	**Continuous,**<br><br>*"*In the past week, on average, how intense was your pain on a 1-10 scale where 0 is "no pain" and 10 is "pain as bad as it could be"?"	**Continuous,** 0-10 scale	Gurung et al (2015) (8)<br>De Zoete et al (2020) (11)<br>Hahne et al (2017) (12)<br>Holden et al (2023)(13)	Greater baseline pain > Lower baseline pain
Baseline disability level	**Continuous**,<br><br>"Roland-Morris Disability Questionnaire"	**Continuous**, 0-24 scale	Gurung et al (2015) (8)<br>Hee et al (2021) (14)<br>Holden et al (2023)(13)	Greater baseline disability (i.e. poorer physical function) > Lower baseline disability
Back beliefs	**Continuous,**<br><br>Back Beliefs Questionnaire	**Continuous,** 9-45 scale	Cashin et al (2023) (15)<br>Chen et al (2023) (16)	Unhelpful beliefs about back pain consequences > helpful beliefs about back pain consequences

Kinesio-phobia	**Continuous**, Tampa Scale of Kinesiophobia	**Continuous**, 17-68 scale	Murillo et al (2023) (17)	Higher kinesiophobia > lower kinesiophobia
Pain catastrophising	**Continuous**, Pain Catastrophizing Scale	**Continuous**, 0-52 scale	Cashin et al (2023) (15)	Higher pain catastrophising > lower levels of pain catastrophising
Pain Self efficacy	**Continuous,**<br><br>Pain Self-Efficacy Questionnaire	**Continuous**, 0-60 scale	Roseen et al (2021) (18)<br>Cashin et al (2023) (15)<br>Chen et al (2023) (16)	Higher pain self-efficacy > lower pain self-efficacy
Back perception	**Continuous**,<br><br>Fremantle Back Awareness Questionnaire	**Continuous**, 0-36 scale	Hagen et al (2005) (19)<br>Wand et al (2016) (20)	Higher levels of body perception disturbance > lower levels of body perception disturbance

Statistical analysis

Baseline demographic and clinical characteristics will be summarized using counts and percentages for categorical variables and means and standard deviations or median and interquartile range for continuous variables.

We will conduct a formal moderation analysis using a test for statistical interaction (21, 22). We will use separate linear regression models to evaluate each potential moderator variable against each outcome at each follow-up timepoint by incorporating a group x potential moderator interaction term. Specifically, we will build each model using a term for outcome (pain intensity or disability), group allocation, potential moderator, and an interaction term for group x moderator. We will estimate the effect size and 95% confidence interval, and report these alongside the p-value, for each group x moderator interaction. Effect sizes for dichotomous moderators will be interpreted as the effect of the intervention, relative to the comparison, in participants with the baseline moderator compared to those without. Effect sizes for continuous moderators will be interpreted as the effect of the intervention, relative to the comparison, for a single unit change in the continuous moderator. We will consider p-values <0.2 as hypothesis generating and p-values of <0.05 as significant. All statistical analyses will be conducted using R [version 3.6.1 or higher].

Missing data

We will assess the proportion and patterns of missing moderator and outcome data. We will conduct all analyses on complete cases if the proportion of missing data is less than 5% for any of the moderators or outcome. If missing data exceeds 5%, we will use multiple imputations by chain equations to impute 10 datasets using the 'mice' package.

References

1. Bagg MK, Wand BM, Cashin AG, Lee H, Hübscher M, Stanton TR, et al. Effect of Graded Sensorimotor Retraining on Pain Intensity in Patients With Chronic Low Back Pain: A Randomized Clinical Trial. JAMA. 2022;328(5):430-9.
2. Barazzetti L, Garcez A, Freitas Sant'Anna PC, Souza de Bairros F, Dias-da-Costa JS, Anselmo Olinto MT. Does sleep quality modify the relationship between common mental disorders and chronic low back pain in adult women? Sleep Medicine. 2022;96:132-9.
3. Nijs J, Mairesse O, Neu D, Leysen L, Danneels L, Cagnie B, et al. Sleep Disturbances in Chronic Pain: Neurobiology, Assessment, and Treatment in Physical Therapist Practice. Physical Therapy. 2018;98(5):325-35.
4. Haack M, Simpson N, Sethna N, Kaur S, Mullington J. Sleep deficiency and chronic pain: potential underlying mechanisms and clinical implications. Neuropsychopharmacology. 2020;45(1):205-16.
5. Klyne DM, Hall M. Is sleep the new treatment for pain? Two issues need resolving before deciding. Sleep. 2024;47(6).
6. Venter M, Grotle M, Øiestad BE, Aanesen F, Tingulstad A, Rysstad T, et al. Treatment Effect Modifiers for Return-to-Work in Patients With Musculoskeletal Disorders. J Pain. 2024;25(9):104556.
7. Beneciuk JM, Hill JC, Campbell P, Afolabi E, George SZ, Dunn KM, et al. Identifying Treatment Effect Modifiers in the STarT Back Trial: A Secondary Analysis. J Pain. 2017;18(1):54-65.
8. Gurung T, Ellard DR, Mistry D, Patel S, Underwood M. Identifying potential moderators for response to treatment in low back pain: A systematic review. Physiotherapy. 2015;101(3):243-51.
9. Beneciuk JM, George SZ, Patterson CG, Smith CN, Brennan GP, Wegener ST, et al. Treatment effect modifiers for individuals with acute low back pain: secondary analysis of the TARGET trial. Pain. 2023;164(1):171-9.
10. Hayden JA, Wilson MN, Stewart S, Cartwright JL, Smith AO, Riley RD, et al. Exercise treatment effect modifiers in persistent low back pain: an individual participant data meta-analysis of 3514 participants from 27 randomised controlled trials. Br J Sports Med. 2020;54(21):1277-8.
11. de Zoete A, de Boer MR, Rubinstein SM, van Tulder MW, Underwood M, Hayden JA, et al. Moderators of the Effect of Spinal Manipulative Therapy on Pain Relief and Function in Patients with Chronic Low Back Pain: An Individual Participant Data Meta-analysis. Spine (Phila Pa 1976). 2021;46(8):E505-e17.
12. Hahne AJ, Ford JJ, Richards MC, Surkitt LD, Chan AYP, Slater SL, et al. Who Benefits Most From Individualized Physiotherapy or Advice for Low Back Disorders? A Preplanned Effect Modifier Analysis of a Randomized Controlled Trial. Spine (Phila Pa 1976). 2017;42(21):E1215-e24.
13. Holden MA, Hattle M, Runhaar J, Riley RD, Healey EL, Quicke J, et al. Moderators of the effect of therapeutic exercise for knee and hip osteoarthritis: a systematic review and individual participant data meta-analysis. The Lancet Rheumatology. 2023;5(7):e386-e400.
14. Hee SW, Mistry D, Friede T, Lamb SE, Stallard N, Underwood M, et al. Identification of subgroup effect with an individual participant data meta-analysis of randomised controlled trials of three different types of therapist-delivered care in low back pain. BMC Musculoskelet Disord. 2021;22(1):191.

15. Cashin AG, Lee H, Wand BM, Bagg MK, O'Hagan ET, Rizzo RRN, et al. Mechanisms of education and graded sensorimotor retraining in people with chronic low back pain: a mediation analysis. Pain. 2023;164(12):2792-800.
16. Chen JA, Anderson ML, Cherkin DC, Balderson BH, Cook AJ, Sherman KJ, et al. Moderators and Nonspecific Predictors of Treatment Benefits in a Randomized Trial of Mindfulness-Based Stress Reduction vs Cognitive-Behavioral Therapy vs Usual Care for Chronic Low Back Pain. The Journal of Pain. 2023;24(2):282-303.
17. Murillo C, Galán-Martín M, Montero-Cuadrado F, Lluch E, Meeus M, Loh WW. Reductions in kinesiophobia and distress after pain neuroscience education and exercise lead to favourable outcomes: a secondary mediation analysis of a randomized controlled trial in primary care. Pain. 2023;164(10):2296-305.
18. Roseen EJ, Gerlovin H, Felson DT, Delitto A, Sherman KJ, Saper RB. Which Chronic Low Back Pain Patients Respond Favorably to Yoga, Physical Therapy, and a Self-care Book? Responder Analyses from a Randomized Controlled Trial. Pain Med. 2021;22(1):165-80.
19. Hagen EM, Svensen E, Eriksen HR. Predictors and Modifiers of Treatment Effect Influencing Sick Leave in Subacute Low Back Pain Patients. Spine. 2005;30(24).
20. Wand BM, Catley MJ, Rabey MI, O'Sullivan PB, O'Connell NE, Smith AJ. Disrupted Self-Perception in People With Chronic Low Back Pain. Further Evaluation of the Fremantle Back Awareness Questionnaire. J Pain. 2016;17(9):1001-12.
21. Pincus T, Miles C, Froud R, Underwood M, Carnes D, Taylor SJ. Methodological criteria for the assessment of moderators in systematic reviews of randomised controlled trials: a consensus study. BMC Med Res Methodol. 2011;11:14.
22. Sun X, Briel M, Walter SD, Guyatt GH. Is a subgroup effect believable? Updating criteria to evaluate the credibility of subgroup analyses. Bmj. 2010;340:c117.
