# Supplementary material for: Effect Modifiers of Graded Sensorimotor Retraining for Chronic Low Back Pain: A Secondary Analysis of the RESOLVE Randomized Trial
Source: JAMA Netw Open. 2026 Jan 13;9(1):e2552787. doi: 10.1001/jamanetworkopen.2025.52787 (PMC12801085; doi:10.1001/jamanetworkopen.2025.52787)
Supplement: Supplement 2. — Data Sharing Statement [file jamanetwopen-e2552787-s002.pdf]

# Data Sharing Statement

Venter. Effect Modifiers of Graded Sensorimotor Retraining for Chronic Low Back Pain. *JAMA Netw Open*. Published January 13, 2026. doi:10.1001/jamanetworkopen.2025.52787

**Data**
**Additional Information:** ANZCTR identifier: ACTRN12615000610538
**Data available:** Yes
**Data types:** Deidentified participant data
**How to access data:** Individual participant data and analytic code that underlie the results reported in this article, after deidentification (text, tables, and figures) will be made available beginning 3 months and ending 5 years following the article publication to researchers who provide a methodologically sound proposal. Proposals should be directed to a.cashin@neura.edu.au to gain access. Data requestors will need to sign a data access agreement.
**When available:** With publication

**Supporting Documents**
**Document types:** None

**Additional Information**
**Who can access the data:** Researchers who provide a methodologically sound proposal
**Types of analyses:** Any purpose
**Mechanisms of data availability:** Data requestors will need to sign a data access agreement
